# Elevated One-Hour Post-Load Glucose Is Independently Associated with Albuminuria: A Cross-Sectional Population Study

**DOI:** 10.3390/jcm11144124

**Published:** 2022-07-15

**Authors:** Anni Saunajoki, Juha Auvinen, Aini Bloigu, Jouko Saramies, Jaakko Tuomilehto, Hannu Uusitalo, Esko Hussi, Henna Cederberg-Tamminen, Kadri Suija, Sirkka Keinänen-Kiukaanniemi, Markku Timonen

**Affiliations:** 1 Center for Life Course Health Research, University of Oulu, 90220 Oulu, Finland; juha.auvinen@oulu.fi (J.A.); abloigu@outlook.com (A.B.); jouko.saramies@fimnet.fi (J.S.); kadri.suija@ut.ee (K.S.); sirkka.keinanen-kiukaanniemi@oulu.fi (S.K.-K.); markku.timonen@oulu.fi (M.T.); 2 Medical Research Center Oulu, Oulu University Hospital and University of Oulu, 90220 Oulu, Finland; 3 South Karelia Social and Health Care District, 53130 Lappeenranta, Finland; hussiesko@gmail.com; 4 Department of Public Health and Welfare, Finnish Institute for Health and Welfare, 00271 Helsinki, Finland; jaakko.tuomilehto@helsinki.fi; 5 Diabetes Research Group, King Abdulaziz University, Jeddah 22254, Saudi Arabia; 6 Department of Ophthalmology, Faculty of Medicine and Health Technology, Tampere University, 33014 Tampere, Finland; hannu.uusitalo@tuni.fi; 7 Tays Eye Centre, Tampere University Hospital, 33014 Tampere, Finland; 8 Department of Endocrinology, Abdominal Center, Helsinki University Hospital, 00290 Helsinki, Finland; henna.cederberg-tamminen@hus.fi; 9 Institute of Family Medicine and Public Health, Faculty of Medicine, University of Tartu, 50411 Tartu, Estonia; 10 Healthcare and Social Services of Selänne, 98530 Pyhäjärvi, Finland; 11 Unit of General Practice, Oulu University Hospital, 90220 Oulu, Finland

**Keywords:** albuminuria, one-hour post-load glucose, oral glucose tolerance test, prediabetic state, type 2 diabetes

## Abstract

The purpose of this study was to examine and compare the associations between albuminuria and fasting (FPG), 1 h post-load (1 h PG) and 2 h post-load plasma glucose (2 h PG) in an oral glucose tolerance test (OGTT). A total of 496 people free of known diabetes (mean age 72 years) participated in the examinations including the OGTT with plasma glucose measurements at 0, 1, and 2 h and levels of HbA1c. Albuminuria was determined by the urinary albumin-to-creatinine ratio and was defined as ≥3.0 mg/mmol. Compared with those without albuminuria, participants with albuminuria had significantly higher 1 h PG and 2 h PG levels, but not FPG or HbA1c levels. An elevated 1 h PG increased the estimated odds ratio of albuminuria more than three times in people with prediabetic 1 h PG (8.6–11.5 mmol/L: OR 3.60; 95% CI 1.70–7.64) and diabetic 1 h PG (≥11.6 mmol/L: OR 3.05; 95% CI 1.29–7.23). After adjusting for blood pressure and age, the association of elevated 1 h PG with albuminuria remained significant. Prediabetic or diabetic FPG, 2 h PG, or HbA1c did not have a statistically significant association with albuminuria. These findings suggest that 1 h PG seems to be the best glycemic parameter and is useful in recognizing persons with an elevated risk of early kidney disease due to hyperglycemia.

## 1. Introduction

Type 2 diabetes is one of the major global health concerns causing long-term complications [1]. Until now, type 2 diabetes in asymptomatic people has been diagnosed based on fasting plasma glucose (FPG), HbA1c or 2 h post-load plasma glucose (2 h PG) during the oral glucose tolerance test (OGTT) [2]. An impaired 2 h PG, with a normal FPG and HbA1c, is an established risk factor for developing type 2 diabetes in later life, and it independently predicts cardiovascular disease and all-cause mortality [3,4]. It is recommended to also implement disease prevention strategies in people with impaired glucose tolerance [5,6,7]. Despite the disease predictive abilities of 2 h PG, previous studies suggested reducing the conventional 2 h OGTT to a 1 h OGTT to make the diagnosis of diabetes and other disorders of glucose regulation more efficient and customer-friendly [8,9,10].

Diabetic kidney disease (DKD) is one of the most frequent and severe complications of type 2 diabetes. From a public health perspective, the overall healthcare costs and individual burden of DKD are extraordinarily high [11]. Thus, there is a need for the early identification of people with an increased risk of DKD, which was first determined by the presence of albuminuria. An increasing number of studies highlighted that compared with 2 h PG, elevated 1 h post-load glucose (1 h PG) has at least a similar predictive value for future type 2 diabetes [9] as well as microvascular [12,13] and macrovascular complications [9,14,15]. To the best of our knowledge, there is only one study that suggested that participants with elevated 1 h PG (≥8.6 mmol/L) had significantly higher albuminuria levels [16].

To evaluate whether elevated 1 h PG is useful in recognizing people at high risk for albuminuria, we examined the association between 1 h PG and albuminuria in an unadjusted model and after adjustment for other putative risk factors for albuminuria. Furthermore, we assessed whether FPG, 1 h PG, 2 h PG, and HbA1c differed in their abilities to identify people at risk of albuminuria in a cross-sectional population-based study.

## 2. Materials and Methods

### 2.1. Study Population

The study population consisted of participants born between 1933 and 1956 and living in the municipality of Savitaipale in a rural area of Eastern Finland. Originally, the study was designed to address the risk factors, prevalence and incidence of abnormal glucose metabolism and other non-communicable diseases. The target population included 1508 participants, and a total of 1168 agreed to participate (attendance rate: 77.5%). The baseline survey was carried out in 1996–1999, and the follow-up surveys were conducted 10 and 22 years after. The data include questionnaire, clinical, and laboratory assessments as well as data from several national health and population registers. The study population and selection criteria were previously described in detail [17]. Participants with incomplete glucose data (*n* = 17) were excluded, yielding a baseline study sample of 1151 participants. The present cross-sectional analyses were performed with the data available at the 22-year follow-up examination (in the years 2018–2019). After the exclusion of participants with incomplete follow-up data due to death (*n* = 245), non-participation (*n* = 269), or a missing or invalid measurement (*n* = 141), 496 individuals were included in the final study population. The study was conducted according to the Declaration of Helsinki and approved by the Ethics Review Board of the South Karelia Hospital District. The Research Ethics Committee of the University of Helsinki approved the research (HUS/2203/2018). Written informed consent was obtained from all subjects involved in the study.

### 2.2. The Definition of the Variables

A standard 75 g OGTT was conducted after 12 h of fasting in participants without a history of diabetes and with FPG < 8 mmol/L. Glucose values were determined at 0, 60, and 120 min after glucose intake. A diabetes diagnosis was made according to the criteria issued by the World Health Organization (WHO) which are as follows: impaired fasting glucose (IFG) as FPG of 6.1 to 6.9 mmol/L and 2 h under 7.8 mmol/L; impaired glucose tolerance (IGT) as fasting glucose under 7.0 mmol/L and 2 h of 7.8 to 11.0 mmol/L; type 2 diabetes as fasting glucose of 7.0 mmol/L or more or 2 h of 11.1 mmol/L or more [18]. An invited expert panel suggested that concentrations of glycated hemoglobin (HbA1c) of 6.0–6.4 % (42.0–47.0 mmol/mol) may be used as the cut-off point for prediabetes and 6.5% (48.0 mmol/mol) and over for diabetes [19]. A large population-based study suggested that 1 h PG ≥ 8.6 mmol/L may be used as a criterion for prediabetes [20]; the current meta-analysis had an optimal cutoff point of 1 h PG ≥ 11.6 mmol/L, which may be used to diagnose type 2 diabetes [21]. Albuminuria was determined by the urinary albumin-to-creatinine ratio (U-Alb/Crea) from a random spot urine collection and was defined as ≥3.0 mg/mmol [22]. Plasma total cholesterol, triglycerides, high-density lipoprotein (HDL) and low-density lipoprotein (LDL) were determined as previously described [17].

Weight, height, and blood pressure were measured in all participants by the study nurse. Waist circumference was measured between the lowest rib and iliac crest without clothing. Body mass index (BMI) was calculated as weight (kg) divided by height squared (m^2^). For the calculation of BMI, height measured at baseline was also used in the 22-year follow-up. Systolic and diastolic blood pressure were measured using an automatic sphygmomanometer (Omron M4-I meter), with the participant in a seated position after 15 min of rest; the averages of the two systolic and diastolic blood pressure measurements were used for the analysis. Hypertension was defined as systolic and diastolic blood pressures of above 140 and/or 90 mmHg or the use of blood-pressure lowering drugs.

### 2.3. Statistical Analysis

Differences in background characteristics were examined with Student’s *t*-test for continuous variables and Pearson’s chi-square test for categorical variables. We used logistic regression to estimate the association between albuminuria and different glucose measurements (categorized as described above). Blood pressure (categories < 140/90 and ≥140 and/or 90 mmHg) and age (continuous variable) were used as adjusting variables. Estimates are presented as odds ratios (OR) with 95% confidence intervals (95% CI). The receiver operating characteristic (ROC) analysis was used to compare the discriminatory ability of FPG, 1 h PG and 2 h PG to detect albuminuria. Analyses were carried out with IBM SPSS Statistics 26.0 (IBM Corp.: Armonk, NY, USA). A *p*-value < 0.05 was considered statistically significant.

## 3. Results

Table 1 describes the characteristics of the study population with and without albuminuria. The mean age of the participants was 72.2 (standard deviation (SD) 6.3) years, and 42.7% (212/496) were men. The prevalence of albuminuria was 10.3% (51/496) and the median (25th to 75th percentile) of the urine albumin-to-creatinine ratio was 7.0 (3.9–10.9) mg/mmol. Among participants with albuminuria, the mean values of 1 h PG, 2 h PG, systolic blood pressure, and age were significantly higher than in participants without albuminuria (*p* < 0.05 for all comparisons). By contrast, no significant difference was found for BMI, waist circumference, FPG, HbA1c, diastolic blood pressure or plasma cholesterol measurements between the participants with and without albuminuria. Based on the results of FPG (categorized as ≤6.0, 6.1–7.0 and ≥7.0 mmol/L), 1 h PG (categorized as <8.6, 8.6–11.5 and ≥11.6 mmol/L), 2 h PG (categorized as <7.8, 7.8–11.0 and ≥11.1 mmol/L) and HbA1c (categorized as <6.0% (42.0 mmol/mol), 6.0–6.4% (42.0–47.0 mmol/mol) and ≥6.5% (48 mmol/mol)), participants were classified into the following three categories: normoglycemia, prediabetes and diabetes. Participants with an increased 1 h PG had a significantly higher prevalence of albuminuria (*p* = 0.002), but such an association was not seen with FPG, 2 h PG, or HbA1c levels. Additionally, the prevalence of hypertension was significantly higher in people with albuminuria than in those without (Table 1).

**Figure 1:**
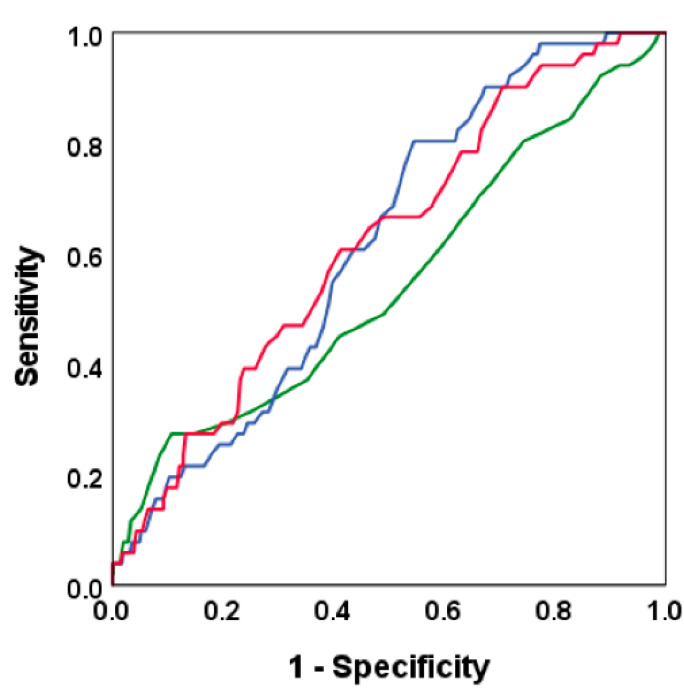
Receiver Operating Characteristic (ROC) curves illustrate the ability of fasting plasma glucose (FPG), 1 h post-load glucose (1 h PG) and 2 h post-load glucose (2 h PG) to detect albuminuria. The green curve indicates FPG (area under the curve (AUC) 0.55; 95% CI 0.46–0.64), blue 1 h PG (AUC 0.62; 95% CI 0.55–0.69), and red 2 h PG (AUC 0.62; 95% CI 0.54–0.70) levels.

**Figure 2:**
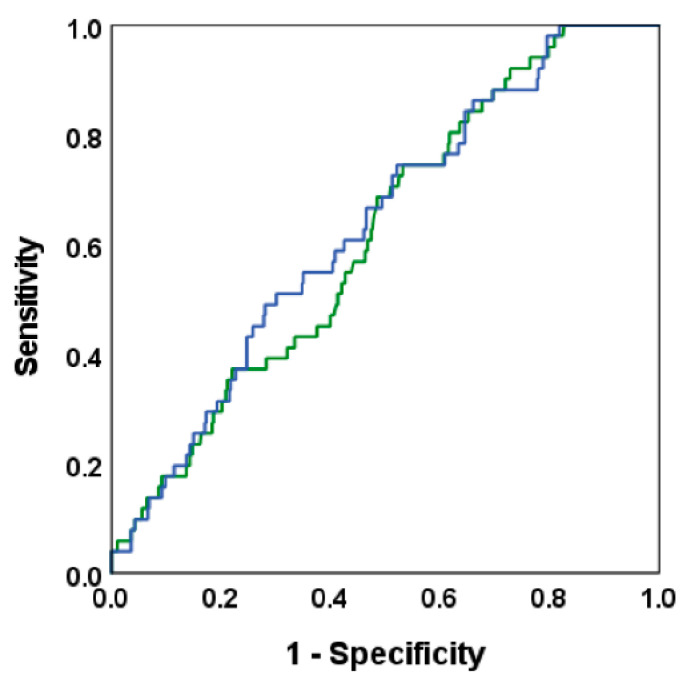
Receiver Operating Characteristic (ROC) curves represent two models to detect albuminuria: the first model (green line) with fasting plasma glucose and 1 h post-load glucose (area under the curve (AUC) 0.62; 95% CI 0.54–0.69), and the second model (blue line) with the first model plus 2 h post-load glucose (AUC 0.63; 95% CI 0.56–0.71, *p* = 0.21).

Using the ROC curve to detect albuminuria, we found that the areas under the curve (AUC) for FPG (AUC 0.55; 95% CI 0.46–0.64), 1 h PG (AUC 0.62; 95% CI 0.55–0.69) and 2 h PG (AUC 0.62; 95% CI 0.54–0.70) did not differ significantly (Figure 1). The optimal cut-off points for detecting albuminuria were 5.9 mmol/L for FPG, 8.7 mmol/L for 1 h PG and 7.3 mmol/L for 2 h PG. Because the cut-off points were close to the diagnostic criteria of prediabetes, we performed the logistic regression analyses using diagnostic threshold values. Furthermore, we assessed whether 1 h OGTT including FPG, and 1 h PG had a similar ability to detect albuminuria compared to 2 h OGTT including FPG, 1 h PG and 2 h PG, using the optimal cut-off points for glucose values based on ROC analyses. As shown in Figure 2, the AUC for the combination of FPG and 1 h PG levels was 0.62 (95% CI 0.54–0.69), and the addition of 2 h PG to this model did not increase the AUC in a statistically significant manner (AUC 0.63; 95% CI 0.56–0.71, *p* = 0.21) (Figure 2).

**Figure 3:**
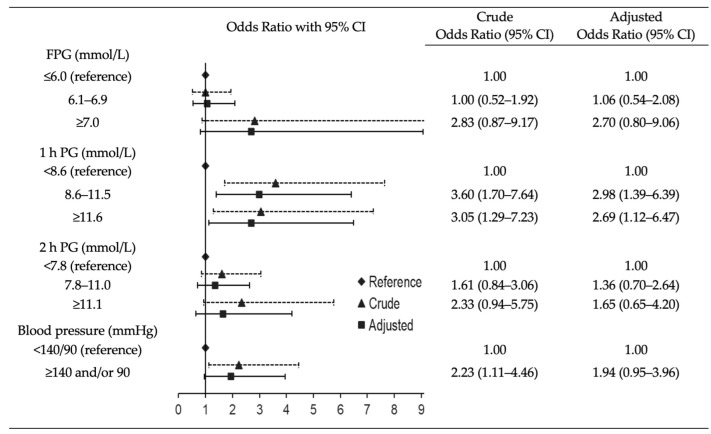
Crude and adjusted odds ratios (95% CI) of albuminuria with fasting plasma glucose, 1 h post-load glucose, 2 h post-load glucose and blood pressure. Dashed line with triangle represents crude values and continuous line with square represents adjusted values. Glucose values were adjusted with blood pressure and age (continuous); blood pressure was adjusted with 1 h post-load glucose and age (continuous).

Furthermore, a logistic regression analysis was performed to examine the association between albuminuria and the glucose status, separately with FPG (categorized as ≤6.0, 6.1–6.9 and ≥7.0 mmol/L), 1 h PG (categorized as <8.6, 8.6–11.5 and ≥11.6 mmol/L) and 2 h PG (categorized as <7.8, 7.8–11.0 and ≥11.1 mmol/L). The ORs with 95% CI were calculated and the analyses were adjusted with the variables that were statistically significant in the t-test and chi-square test (Table 1). Figure 3 shows that elevated 1 h PG with cut-off points of 8.6–11.5 mmol/L and ≥11.6 mmol/L was associated with albuminuria (OR 3.60; 95% CI 1.70–7.64 and OR 3.05; 95% CI 1.29–7.23, respectively), whereas elevated FPG and 2 h PG levels had non-significant estimated ORs. After adjusting for blood pressure (categorized as <140/90 and ≥140 and/or 90 mmHg) and age, the association between 1 h PG and albuminuria remained statistically significant (OR 2.98; 95% CI 1.39–6.39 for 8.6–11.5 mmol/L and OR 2.69; 95% CI 1.12–6.47 for ≥11.6 mmol/L). The unadjusted association between elevated blood pressure and albuminuria was significant (OR 2.23; 95% CI 1.11–4.46), but the association disappeared after adjustment for 1 h PG and age (Figure 3).

## 4. Discussion

In this cross-sectional population-based study, we found for the first time that among people without a history of diabetes, prediabetic and diabetic 1 h PG—but not FPG, 2 h PG or HbA1c—were statistically significantly associated with albuminuria, both in the unadjusted and adjusted model including blood pressure and age. The mean 2 h PG was elevated in people with albuminuria, but prediabetic and diabetic 2 h PG values were not associated with albuminuria. Furthermore, the addition of 2 h PG to the model, including FPG and 1 h PG, did not improve the detection of albuminuria. In our study population, the ability of 1 h PG to identify individuals with albuminuria seemed to be better than that of other indicators of hyperglycemia.

The current study demonstrated a significant and independent association between prediabetic and diabetic 1 h PG and albuminuria. A few studies previously looked at the association between 1 h PG and microalbuminuria levels as well as kidney involvement. One previous study, which was primarily designed to assess the association between 1 h PG and metabolic syndrome and hyperglycemic disorders, stated that participants with normal FPG and 2 h PG, but elevated 1 h PG (≥8.6 mmol/L), had a significantly higher level of microalbuminuria determined by 24 h urine collection compared with participants with normal FPG, 1 h PG and 2 h PG levels. The difference in microalbuminuria levels was also significant when participants with prediabetes and diabetes were included, but the significance disappeared after adjustment with age and gender [16]. Until now, increased 1 h PG has been shown to be associated with a lower estimated glomerular filtration rate (GFR), indicating a 2.61-fold (95% CI 1.01–6.77) risk for diminished GFR compared with participants with normal 1 h PG [23]. GFR generally falls progressively after a further rise in albuminuria.

In addition to the association between 1 h PG and albuminuria, 1 h PG and 2 h PG had a similar ability in detecting albuminuria in the ROC analyses, and the addition of 2 h PG to 1 h OGTT did not improve the ability to recognize albuminuria. Furthermore, in this study, elevated FPG, 2 h PG and HbA1c were not independently associated with significant albuminuria. Recent studies focusing on the association between elevated FPG and/or 2 h PG levels with albuminuria reported contradictory results. While the prospective studies stated that prediabetic FPG is a risk factor of albuminuria, a cross-sectional study found post-challenge hyperglycemia to be more significantly associated with albuminuria than FPG [24,25,26]. Moreover, HbA1c was found to be associated with low-grade albuminuria in middle-aged and elderly Chinese people, irrespective of diabetes status. The determined optimal cut-off point for HbA1c was 5.7% (39 mmol/mol) with a poor AUC of 0.58 (0.56–0.59) [27].

From a pathophysiological point of view, the association between 1 h PG and albuminuria may be explained by several possible factors. One reason could be that both 1 h PG and albuminuria are associated with cardiovascular risk factors including increased blood pressure and an unfavorable lipid profile [14,15,28,29]. Furthermore, elevated 1 h PG is primarily suggested to be caused by the following two main pathophysiological mechanisms: insulin resistance and diminished β-cell function [30]. In line with this, reduced insulin sensitivity appeared to also be associated with a greater risk of albuminuria by inducing glomerular infiltration [31,32]. It should also be kept in mind that elevated 1 h PG can be associated with an unfavorable inflammation profile, which is also a part of the pathogenetic mechanisms of DKD [33,34]. However, the possibility that both elevated 1 h PG and albuminuria occur in parallel as a consequence of similar metabolic disturbances needs to be investigated further.

The main strengths of this study were that we compared the association between albuminuria and elevated FPG, 1 h PG, and 2 h PG levels, while previous research reported the comparison of elevated and normal 1 h PG with microalbuminuria [16,23]. In addition, albuminuria was determined by the albumin/creatinine ratio, which is the recommended tool to detect individuals at high risk of DKD [35]. The limitations of our study include the cross-sectional study design, which cannot provide any information about cause and effect between albuminuria and abnormal glucose metabolism. Further, this study had a relatively small sample size and certain subgroup analyses could not be performed. The generalizability of our results beyond the elderly population may also be limited and our findings need to be replicated in middle-aged populations. Moreover, typical of epidemiological studies, only one urine albumin-creatinine ratio specimen was taken from the participants, which might overestimate the prevalence of albuminuria. Due to the variability of albumin excretion into the urine, two of three specimens collected within a 3- to 6-month period should be abnormal before albuminuria is clinically confirmed [22]. Data on other indicators of kidney function, such as plasma creatinine, were not available.

## 5. Conclusions

We demonstrated that prediabetic and diabetic 1 h PG, but not FPG and 2 h PG, were associated independently with the prevalence of albuminuria. The use of 1 h PG may allow for earlier interventions by recognizing these high-risk people and, thereby, postpone the onset of DKD. These are issues that need to be addressed in the future when considering the clinical usefulness of 1 h PG in the OGTT. There are matters unresolved in the present study that require further investigation. First, we suggest that prospective studies evaluate the ability of 1 h PG to predict the incidence of albuminuria. Additionally, interventional studies are needed that provide information on the clinical implications and cost-effectiveness of the use of 1 h PG in this context.

## Figures and Tables

**Table 1:** Clinical characteristics of the study participants by albuminuria.

	U-Alb/Crea < 3.0	U-Alb/Crea ≥ 3.0	*p* Value
Study population, ***n*** (%)	445 (89.7)	51 (10.3)	
Sex, ***n*** (%)			0.209
Men	186 (87.7)	26 (12.3)	
Women	259 (91.2)	25 (8.8)	
Age (years)	71.8 ± 6.2	75.4 ± 6.4	<0.001
BMI (kg/m^2^)	26.9 ± 4.2	27.5 ± 5.4	0.444
Waist circumference (cm)	92.7 ± 11.9	94.1 ± 13.6	0.424
Men (cm)	97.3 ± 10.7	97.7 ± 13.6	0.870
Women (cm)	89.4 ± 11.6	90.4 ± 12.7	0.693
Fasting plasma glucose (mmol/L)			
Mean	5.8 ± 0.6	6.0 ± 1.0	0.108
≤6.0, ***n*** (%)	303 (68.1)	33 (64.7)	0.187
6.1–6.9, ***n*** (%)	129 (29.0)	14 (27.5)	
≥7.0, ***n*** (%)	13 (2.9)	4 (7.8)	
1 h post-load glucose (mmol/L)			
Mean	9.0 ± 2.7	10.3 ± 2.9	0.001
<8.6, ***n*** (%)	202 (45.4)	10 (19.6)	0.002
8.6–11.5, ***n*** (%)	157 (35.3)	28 (54.9)	
≥11.6, ***n*** (%)	86 (19.3)	13 (25.5)	
2 h post-load glucose (mmol/L)			
Mean	7.3 ± 2.3	8.5 ± 3.5	0.015
<7.8, ***n*** (%)	296 (66.5)	27 (52.9)	0.108
7.8–11.0, ***n*** (%)	116 (26.1)	17 (33.3)	
≥11.1, ***n*** (%)	33 (7.4)	7 (13.7)	
HbA1c (mmol/mol)			
Mean	40.7 ± 5.0	42.0 ± 6.3	0.074
<6.0% (42.0 mmol/mol), ***n*** (%)	255 (57.3)	21 (41.2)	0.085
6.0–6.4 (42.0–47.0 mmol/mol), ***n*** (%)	156 (35.1)	24 (47.1)	
≥6.5% (48 mmol/mol), ***n*** (%)	34 (7.6)	6 (11.8)	
Systolic blood pressure (mmHg)	146 ± 21	155 ± 25	0.005
Diastolic blood pressure (mmHg)	83 ± 10	85 ± 12	0.260
Hypertension			
<140/90 mmHg, ***n*** (%)	169 (38.0)	11 (21.6)	0.021
≥140 and/or 90 mmHg, ***n*** (%)	276 (62.0)	40 (78.4)	
Plasma total cholesterol (mmol/L)	4.9 ± 1.0	4.8 ± 1.0	0.411
Plasma HDL cholesterol (mmol/L)	1.5 ± 0.4	1.4 ± 0.4	0.116
Men (mmol/L)	1.4 ± 0.3	1.3 ± 0.3	0.372
Women (mmol/L)	1.6 ± 0.4	1.6 ± 0.4	0.385
Plasma LDL cholesterol (mmol/L)	3.1 ± 0.9	3.1 ± 1.0	0.947
Plasma triglycerides (mmol/L)	1.2 ± 0.5	1.2 ± 0.7	0.504
Men (mmol/L)	1.1 ± 0.5	1.3 ± 0.8	0.186
Women (mmol/L)	1.2 ± 0.4	1.1 ± 0.4	0.320

Continuous variables are presented as mean ± standard deviation. Categorical variables are presented as counts and percentages (%).

## Data Availability

The datasets generated and/or analyzed during the current study are available from the corresponding author upon reasonable request.
